# A Biopsychosocial Framework for Apathy Following Moderate to Severe Traumatic Brain Injury: A Systematic Review and Meta-analysis

**DOI:** 10.1007/s11065-023-09620-4

**Published:** 2023-12-19

**Authors:** Halle Quang, Travis Wearne, Michaela Filipcikova, Nhi Pham, Nhi Nguyen, Skye McDonald

**Affiliations:** 1 School of Health Sciences and Brain & Mind Centre, University of Sydney, Sydney, Australia; 2 School of Psychology, University of New South Wales, High Street, Kensington, NSW 2033 Australia; 3 Western Sydney University, Sydney, Australia; 4 School of Psychology, Ho Chi Minh City University of Education, Ho Chi Minh City, Vietnam; 5 Ho Chi Minh City University of Social Sciences and Humanities, Ho Chi Minh City, Vietnam

**Keywords:** Motivation, Head injury, Culture, Neuroimaging, Individual, Biopsychosocial

## Abstract

Apathy, the deficit of goal-directed behaviour, is well recognised as one of the most debilitating syndromes after moderate-to-severe traumatic brain injury (TBI). However, mechanisms underlying apathy, or at least factors associated with apathy, are sporadically reported. Based on a biopsychosocial framework, this systematic review and meta-analysis synthesised evidence regarding neurobiological, socio-environmental and individual factors associated with apathy. Our searches identified 21 studies satisfying inclusion and exclusion criteria. Results showed that the majority of work has focused on cognitive dysfunction, TBI-related factors, demographic variables and psychological correlates of apathy, while evidence for neural substrates and socio-cultural and premorbid aspects is scant. Overall, the current literature suggests that TBI-related and patient demographic factors did not contribute to apathy after TBI, whereas complex neurocognitive alterations, socio-environmental and cultural factors as well as patients’ self-related factors may be important components. The evidence points to the multifaceted interplay of certain biopsychosocial contributors to apathy and suggests future investigations of more complex behavioural traits, cultural elements and pre-injury levels to better characterise the aetiology of this detrimental impairment after TBI.

**Supplementary Information:**

The online version contains supplementary material available at 10.1007/s11065-023-09620-4.

## Introduction

Traumatic brain injury (TBI) results in changes to brain functions due an external mechanical force (Maas et al., 2008; Menon et al., 2010). TBI is a major health problem that results in distressing consequences for adults and their families, as well as increased social and economic burden for society (Maas et al., 2008; Nguyen et al., 2013). According to the mechanism of external force, TBI can be classified as (1) closed head injury, which refers to the impact of external forces to the brain usually associated with acceleration and deceleration of brain tissue whereas the skull, dura and brain membrane remain intact; and (2) open or penetrating head injury, which is characterised by the presence of skull fractures and torn dura (McDonald et al., 2013; Murray et al., 1996).

By severity, while there are multiple ways that a TBI can be classified (Peskind et al., 2013), TBI is commonly grouped into mild, moderate and severe levels according to the depth of coma assessed with the Glasgow Coma Scale (GCS) and altered consciousness measured by post-traumatic amnesia (PTA). Mild TBI is identified when patients have GCS score higher than 12 and PTA less than 1 day. Moderate TBI is when GCS is from 9 to 12 with PTA from 1 to 7 days and severe TBI, while a severe TBI is diagnosed if GCS is lower than 8 and PTA is longer than 7 days (Carroll et al., 2004). While global estimates for mild TBI prevalence are much larger, moderate-to-severe brain injuries often lead to numerous and persistent negative impairments, especially devastating neuropsychological and behavioural symptoms (Dewan et al., 2018; Hoofien et al., 2001).

Among the behavioural and neuropsychological symptoms arising after moderate-to-severe TBI, apathy is reported as one of the most common disturbances (Ciurli et al., 2011; Gray et al., 1994). Apathy is the impairment of goal-directed behaviour manifesting in executive, emotional and initiation aspects (Levy & Dubois, 2006; Radakovic & Abrahams, 2018). Executive apathy is the reduction of planning and organisation. Emotional apathy is associated with affective blunting and decreased engagement in social events, while initiation apathy is the impairment of behavioural execution.

Apathy is associated with reduced levels of independence, communication, community integration, and physical and psychological functioning when patients are discharged from hospital (Arnould et al., 2015; Cattelani et al., 2008; Filipčíková et al., 2022; Gray et al., 1994; Green et al., 2022; Hamilton et al., 2015; Mazaux et al., 1997). Patients with apathy can also show reluctance to engage in rehabilitation and resistance to care, making the recovery process of TBI very challenging. Furthermore, apathy can affect the carer’s quality of life by increasing their overall caregiving burden and stress (Arnould et al., 2015; Marsh et al., 1998). Despite the significant impact on patients and their carers, the existing evidence base for the mechanisms underpinning, or at least factors associated with, apathy after TBI is patchy. This is surprising because apathy has somewhat been elaborated in other forms of brain impairments. For example, disruptions to the neural network associated with motivated behaviour have been evident to underlie apathy in stroke (for a review, see Tay et al., 2021) or dementia (Kumfor et al., 2018; Quang et al., 2021b). In TBI at least, an important clinical challenge is to understand the aetiologic complexity of apathy, as the literature has shown that use of a single pharmacological or non-pharmacological measure that only targets one element of the whole picture is not likely to produce positive and sustainable results (Lane‐Brown & Tate, 2009a, b).

Considering apathy as the interaction between multiple factors is not a new concept, as Marin – the pioneering scientist in apathy – put forward the biopsychological approach to apathy in 1991. Marin highlighted the importance of assessing apathy from both medical and environmental perspectives (Marin & Wilkosz, 2005). In TBI, medical factors including neuropathological causes and the loss of sensory or motor functions can contribute to apathy. On the other hand, environmental factors can include the impact of reduced reward or incentive sources, such as a role change in family or institutionalism (Marin & Wilkosz, 2005).

Recently, this framework has been extended and elaborated further. Based on Kales’ model (Kales et al., 2015), which characterises bio-psycho-social components of behavioural and psychological syndromes, Massimo et al. (2018) proposed a similar conceptual model for the specific understanding of apathy in dementia. Components of this model include (1) direct neurobiological disruption and (2) indirect factors including genetics, caregiver and environmental factors that may exacerbate or trigger apathy symptoms. The framework has been formulated based on well-established and also emerging evidence concerning apathy in dementia. It is thought to provide a necessary step forward in the proactive assessment and targeted treatment of apathy. While this framework is equally applicable for apathy after TBI (Anderson et al., 2022), much of the existing research in TBI has also suggested an additional factor – the individual factor (for a review, see Fleming & Ownsworth, 2006; Ponsford, 2013). This factor concerns the patient’s specific characteristics such as pre-injury personality, demographic factors, self-esteem and self-efficacy.

Taken together, a conceptual biopsychosocial framework for understanding the mechanisms of apathy after moderate-to-severe TBI can be productively defined as consisting of three domains: neurobiological, socio-environmental and individual. The purpose of the current study is to systematically review and, where possible, meta-analyse evidence about neurobiological, socio-environmental and individual factors of apathy after TBI. Up to date, a preliminary search (performed on 14 November 2022) of PubMed, Cochrane Database of Systematic Reviews, CINAHL, Joanna Briggs Database of Systematic Reviews, PROSPERO and EPISTEMONIKOS found that there were no recent systematic reviews exploring this precise review objective.

## Methods

This systematic review was conducted according to the Preferred Reporting Items for Systematic Reviews and Meta-analyses (PRISMA) statement (Moher et al., 2009).

### Data Source

We systematically searched the electronic databases PubMed, MEDLINE, PsychINFO, Embase and Scopus. The search was carried out on 21 November 2018, with updates on 30 January 2020 and 22 March 2022 using a consistent method throughout. To capture all available factors associated with apathy, keywords related to **TBI** and **apathy** were utilised, as follows:

- *TBI:* “traumatic brain injury” OR “brain injury” OR “acquired brain injury” OR TBI OR “acquired brain impairment” OR “brain insult” OR “brain trauma” OR “head injury” OR “head trauma”
- *Apathy:* apathy OR amotivation OR anhedonia OR indifference OR “neuropsychiatric symptoms” OR initiation OR apathetic OR “goal-directed behaviour” OR “apathy evaluation scale” OR “apathy scale” OR “neuropsychiatric inventory” OR “apathy inventory” OR “lille apathy rating scale” OR “frontal systems behaviour scale”.

The references were then exported and managed using EndNote X9. We also examined reference lists of reviews and publications that met inclusion criteria and searched the Google Scholar search engine using the names of the individual measures for assessing apathy. Additional studies were also exported and further assessed.

### Study Selection and Eligibility Criteria

The following inclusion criteria were developed and applied to all search strategies and retrieved articles: (1) were original articles, (2) were focused on adult patients with moderate-to-severe TBI defined as age of 16 years or older and using the diagnostic criteria set out in the introduction (Carroll et al., 2004), with (3) presence of apathetic syndrome as examined using a validated approach of measuring apathy, (4) included at least 10 participants, (5) were written in English and (6) published in peer-reviewed journals from 1980 to present. Articles were excluded if they (1) used mixed samples, paediatric or animal samples; (2) included patients with other neurological impairment (co-morbid disorders of dementia, tumour, stroke etc.), patients with cognitive impairment arising from a non-TBI aetiology, patients with psychiatric disorders other than depression and anxiety; and (3) examined only mild traumatic brain injury or concussion. We also excluded book chapters, books sections, letters, editorials, conference proceedings, case studies and review papers.

Articles were selected based on a two-step approach. Titles and abstracts were screened based on the determined criteria. Full texts of selected studies were then obtained for further evaluation.

### Quality Assessment

The National Institutes of Health (NIH) quality assessment scale for cross-sectional and cohort studies was modified to meet the context of this review and applied to examine the methodological quality of the included studies. We removed two items from the original version (Item 10 – Was the exposure(s) assessed more than once over time? and Item 13 – Was the loss to follow-up after baseline 20% or less?) for cross-sectional studies. We added one item (Item 15 in the modified version – Was a control group included and matched for age, education and gender with the TBI group?) since the inclusion of a control group enables more accurate determination of apathy and its correlations with clinical and neurobiological features after moderate-to-severe TBI, relative to the general population. The final version of the quality assessment tool can be found in Supplementary Material 1. Quality assessments were performed independently by two authors (HQ and MF). Ratings from the two assessors achieved excellent inter-rater reliability (*r* = 0.81, *p* < 0.001). The average of the ratings was reported as the final quality scores, with the highest possible score being 13 for cross-sectional studies and being 15 for cohort studies. Cross-sectional studies were considered of ‘Good’ quality with scores ranging from 10 to 13, ‘Fair’ quality with scores 6–9.5 and ‘Poor’ quality with scores lower than 6. Cohort/longitudinal studies were considered of ‘Good’ quality with scores ranging from 11.5 to 15, ‘Fair’ quality with scores 7–11 and ‘Poor’ quality with scores lower than 7.

### Data Extraction and Synthesis

The information extracted from each full-text article reviewed is reported in seven tables. Table 1 summarises basic characteristics of the included studies and their details about the TBI sample. Table 2 denotes the prevalence and severity of apathy after TBI. Tables 3, 4, 5, 6 and 7 present key results of this review. They include apathy-associated factors organised according to subdomains of the neurobiological, social-environmental and individual aspects. Each of these tables includes Authors & Publication date; Country; Quality score, Apathy measure and, importantly, details relating to each factor (i.e. measure, score, whether the factor has a significant association with apathy). When a duplication of findings occurred in multiple papers with overlapping samples, we selected the article with the largest sample size to report.

**Table 1:** Study characteristics

**Article characteristics**				**TBI group characteristics**						**Apathy measure**
**Article**	**Quality score**	**Country**	**Design**	**Sample size**	**Age (years)**	**Education**	**TBI severity**	**Time since injury**	**Sex** **(% Male)**	
(Andersson & Bergedalen, 2002)	6	Norway	Cross-sectional	53	28.3 ± 9.71 (16–54)	12.3 ± 2.38 (9–19 years)	Severe	12.2 ± 10.06 (2–36 months)	75.5	C-AES
(Andersson et al., 1999)	6.5	Norway	Cross-sectional	30	30.1 SE2.26 (16–64)	12.7 SE0.41	Severe	10.5 SE1.68 (2–34 months)	73.3	C-AES
(Arnould et al., 2015)	6	France	Cross-sectional	68	35.56 ± 13.68 (18–74)	13.10 ± 3.21 (7–20 years)	Severe	38.85 ± 29.47 (3–120 months)	83.8	C-AI
(Arnould et al., 2018b)	9.75	France	Cross-sectional	38	34.24 ± 12.52 (18–65)	13.39 ± 3.16 (7–21 years)	Severe	44.18 ± 38.85 (3–144 months)	85.92	I-IIS
										S-IIS
(Arnould et al., 2018c)	9.75	France	Cross-sectional	34	34.91 ± 13.15 (18–65)	13.50 ± 3.27 (7–21 years)	Severe	55.68 ± 59.27 (4–277 months)	82.4	I-IIS
(Arnould et al., 2018a)	11.25	France	Longitudinal	32	34.78 ± 13.47 (18–65)	13.78 ± 3.16 (7–21 years)	Severe	54.84 ± 60.25 (4–277 months)	84.4	I-IIS
(Bivona et al., 2019)	10	USA	Cross-sectional	30	31.07 ± 13.53	13.1 ± 3.23	Severe	≥ 6 months	73.3	I-NPI
(Cristofori et al., 2019)	10.5	USA	Cross-sectional	132	63.29 ± 2.851	14.56 ± 2.24	Severe	40–45 years	100	S-FrSBe
										I-FrSBe
(De Simoni et al., 2018)	10	UK	Cross-sectional	42	40.6 ± 11.7 (20–65)	–	Moderate to severe	73.1 ± 86.9 (6–366 months)	11.9	S-FrSBe-A
										I-FrSBe-A
										S-LARS
(Diaz et al., 2012)	9.5	Brazil	Longitudinal	33	31.36 ± 11.0	9.9 ± 4.7	Severe	18.4 ± 6 (months)	87.5	S-AES
(Glenn et al., 2002)	8	USA	Cross-sectional	46 (moderate to severe = 21)	43.1 ± 14.9	–	Moderate to severe	43.9 ± 57.7 (months)	70	I-AES
										S-AES
(Hart et al., 2019)	9	USA	Cross-sectional	51	37.5 ± 16.3	13.6 ± 2.5	Moderate to severe	3 months (*n* = 51) 6 and 12 months (retested, *n* = 36)	69	S-FrsBe-A
										I-FrSBe-A
(Hogeveen et al., 2017)	10	USA	Cross-sectional	91	63.38 ± 3.06	14.57 ± 2.13	Penetrating	–	100	I-FrSBe-A
(Kilmer et al., 2006)	8	USA	Cross-sectional	51	38.06 ± 19.08	12.12 ± 2.63	Moderate to severe	1 year – 29 2 years – 22	72	I-NPI
(Knutson et al., 2014)	10.75	USA	Cross-sectional	176	58.44 ± 3.16	14.85 ± 2.55	Penetrating	–	100	I-NPI
(Lane-Brown & Tate, 2009a, b)	9	Australia	Cross-sectional	34	34.36 ± 9.39 (18–56)	–	Severe	80.58 ± 71.64 (8–253 months)	88.24	I-AES
										I-FrSBe-A
(Lengenfelder et al., 2015)	8.5	USA	Cross-sectional	33	40.94 ± 11.08 (18–60)	14.58 ± 2.26	Moderate to severe	93.53 ± 66.49 (months)	–	S-FrSBe-A
										I-FrSBe-A
(Monsalve et al., 2012)	7.5	Spain	Cross-sectional	53	35 ± 14.2 (17–69)	–	Severe	2–8 years (2–4 years *n* = 29, 54.7%, 5–8 years *n* = 24, 45.2%)	84.9	I-NPI
(Navarro-Main et al., 2021)	8.75	Spain	Cross-sectional	40	41.6 (18–75)	–	Moderate to severe	26.2 ± 13.7 (6 months)	82.5	I-AES
(Niemeier et al., 2013)	8	USA	Cross-sectional	101	43.36 ± 19.19	–	Moderate to severe	–	64.4	S-FrSBe-A
										I-FrSBe-A
(Quang et al., 2021a)	11	Vietnam	Cross-sectional	61	34.92 ± 11.86	8.87 ± 4.09	Moderate to severe	29.36 ± 10.81 (months)	88.52	S-DAS
										I-DAS

**Table 2:** Prevalence and severity of apathy following moderate-to-severe traumatic brain injury

**Article characteristics**			**Apathy measure**	**Prevalence of apathy after TBI**		**Severity of apathy after TBI**	
**Article**	**Quality score**	**Country**		**Prevalence (%)**	**Cut-off score**	**Mean** ± **SD**	**Significant impairment compared to controls?**
(Andersson & Bergedalen, 2002)	6	Norway	C-AES	62.3	≥ 34	37.0 ± 7.59	No control group
(Navarro-Main et al., 2021)	8.75	Spain	I-AES			24.1 (range: 6–43)	No control group
(Lane-Brown & Tate, 2009a, b)	9	Australia	I-AES	69	≥ 37	40.7 ± 9.8	No control group
			I-FrSBe-A	72	*t*-score ≥ 65	75 ± 19.4	No control group
(Diaz et al., 2012)	9.5	Brazil	S-AES			28.4 ± 14.9	No control group
(Glenn et al., 2002)	8	USA	I-AES			39.4 ± 9.1	No control group
			S-AES			37.3 ± 8.8	No control group
(Hogeveen et al., 2017)	10	USA	I-FrSBe-A	vmPFC: 48 dmPFC: 30 Other TBI: 31	*t*-score ≥ 65	62.69 ± 18.84	Yes
(Lengenfelder et al., 2015)	8.5	USA	S-FrSBe-A	43	*t*-score ≥ 65		
			I-FrSBe-A	35	*t*-score ≥ 65		
(Hart et al., 2019)	9	USA	S-FrsBe-A			60.0 ± 17.5	Yes
			I-FrSBe-A			72.2 ± 20.3	Yes
(De Simoni et al., 2018)	10	UK	S-FrSBe-A			35.8 ± 10.7	No control group
			I-FrSBe-A			30.5 ± 8.4	No control group
			S-LARS			− 23.1 ± 10.3	Yes
(Niemeier et al., 2013)	8	USA	S-FrSBe-A			30.53 ± 8.84	No control group
			I-FrSBe-A			32.69 ± 9.47	No control group
(Arnould et al., 2018b)	9.75	France	I-IIS			23.34 ± 6.58	Yes
			S-IIS			21.16 ± 4.77	Yes
(Kilmer et al., 2006)	8	USA	I-NPI			0.83 ± 1.03	No control group
(Monsalve et al., 2012)	7.5	Spain	C-NPI	49	Yes/no		
(Quang et al., 2021a)	11	Vietnam	S-DAS			Executive apathy: 6.16 ± 4.18 Emotional apathy: 10.51 ± 4.12 Initiation apathy: 15.54 ± 4.95	No (control more impaired than TBI) No Yes
			I-DAS			Executive apathy: 8.69 ± 5.30 Emotional apathy: 11.38 ± 4.39 Initiation apathy: 17.02 ± 5.07	Yes Yes Yes

**Table 3:** Cognitive dysfunction associated with apathy after TBI

**Article characteristics**		**Apathy measure**	**Cognitive correlates**			
**Article**	**Quality score**		**Cognitive domain**	**Measure**	**Mean ± SD**	**Significant effect**
(Andersson & Bergedalen, 2002)	6	C-AES	General cognition – verbal skills	Wechsler Adult Intelligence Scale – Similarities and Comprehension	47.6 ± 10.3	No
			General cognition – non-verbal skills	Wechsler Adult Intelligence Scale – Block Design, Picture Completion and Picture Arrangement	44.6 ± 9.5	No
			Learning	Ten Word List from Luria’s Neuropsychologic Assessment – Acquisition Subtest	33.0 ± 15.2	Yes (*r* = − 0.50)
			Learning	California Verbal Learning Test – Total trials and Delayed recall		
			Processing speed – information	Trail Making Tests A and B	38.8 ± 10.3	No
				Wechsler Adult Intelligence Scale – Digit Symbol		
				Symbol Digit Modalities Test – Oral and Written Version		
			Generalisability and flexibility	Wisconsin Card Sorting Test – correct responses and perseverative responses (computer version)	45.9 ± 11.6	Yes (*r* = − 0.38)
			Attention	Wechsler Adult Intelligence Scale – Digit Span	45.5 ± 8.8	No
				Knox Cube Test		
			Processing speed – Motor	Grooved Pegboard Test	38.6 ± 11.9	No
				Finger Tapping Test		
(Arnould et al., 2018b)	9.75	I-IIS	Learning	California Verbal Learning Test – Attention Span, Learning Efficiency, Delayed Memory and Inaccurate Memory	6.03 ± 1.95	Yes (*r* = − 48)
					32.59 ± 4.22	
					11.22 ± 3.39	
					3.63 ± 3.9	
			Planning	Meeting Preparation Task	1.34 ± 1.53	No
			Attention	Trail Making Test	55.32 ± 36.56	
			Processing speed	Simple Reaction Time Task	0.25 ± 0.1	
		S-IIS	Learning	California Verbal Learning Test – Attention Span, Learning Efficiency, Delayed Memory and Inaccurate Memory	6.03 ± 1.95	No
					32.59 ± 4.22	
					11.22 ± 3.39	
					3.63 ± 3.9	
			Planning	Meeting Preparation Task	1.34 ± 1.53	No
			Attention	Trail Making Test	55.32 ± 36.56	
			Processing speed	Simple Reaction Time Task	0.25 ± 0.1	
(Arnould et al., 2018c)	9.75	I-IIS	Learning	California Verbal Learning Test – Attention Span, Learning Efficiency, Delayed Memory and Inaccurate Memory	6.15 ± 1.96	No
					32.60 ± 4.12	Yes (*r* = − 0.37)
					11.15 ± 3.40	Yes (*r* = − 0.46)
					3.81 ± 4.07	No
			Planning	Meeting Preparation Task	1.32 ± 1.36	Yes (*r* = 0.36)
			Attention	Trail Making Test	50.71 ± 30.03	No
			Processing speed	Simple Reaction Time Task	0.26 ± 0.10	No
(Arnould et al., 2018a)	11.25	I-IIS	Learning	TAP 2-back (omission)	2.66 ± 2.55	Yes vs apathy scores at time 2 (*r* = − 0.54)
				California Verbal Learning Test – Attention Span, Learning Efficiency, Delayed Memory and Inaccurate Memory	6.19 ± 2.02	
					32.87 ± 4.01	
					11.29 ± 3.43	
					3.48 ± 4.08	
			Planning	Meeting Preparation Task	1.28 ± 1.4	Yes vs apathy scores at Time 2 (*r* = 0.43, *p* < 0.01)
			Processing speed	Simple Reaction Time Task	0.26 ± 0.10	No
			Attention	Trail Making Test	51.28 ± 30.89	No
(Bivona et al., 2019)	10	I-NPI	Generalisability and flexibility	Wisconsin Card Sorting Test	4.33 ± 2.26	Yes (*r* = − 0.384)
					21.58 ± 23.89	Yes (*r* = 0.434)
(Knutson et al., 2014)	10.75	I-NPI	Working memory	Wechsler Memory Scale – Working memory primary index percentile score	49.98 ± 28.22	No
			Postinjury intelligence	Armed Forces Qualification Test	53.55 ± 24.50	Yes (*r* = 0.17)
(Lane-Brown & Tate, 2009a, b)	9	I-AES	Learning	Rey Auditory Verbal Learning Test	6.4 ± 3.9	No
			Generalisability and Flexibility	Thurstone Word Fluency Test	31.4 ± 13.8	No
		I-FrSBe-A	Learning	Rey Auditory Verbal Learning Test	6.4 ± 3.9	No
			Generalisability and Flexibility	Thurstone Word Fluency Test	31.4 ± 13.8	No
(Lengenfelder et al., 2015)	8.5	S-FrSBe-A	Attention	Digit Span – Scaled Score	9.19 ± 2.65	Yes (*r* = − 0.447)
			Generalisability and flexibility	Delis–Kaplan Executive Function System Verbal Fluency	33.08 ± 11.23	No
			Generalisability and flexibility	Wisconsin Card-Sorting Test	15.57 ± 12.87	No
		I-FrSBe-A	Attention	Digit Span – Scaled Score	11.68 ± 2.36	No
			Generalisability and flexibility	Delis–Kaplan Executive Function System Verbal Fluency	33.08 ± 11.23	No
			Generalisability and flexibility	Wisconsin Card Sorting Test	10.74 ± 10.84	No
(Quang et al., 2021a)	11	I-DAS	General cognition	Montreal Cognitive Assessment	17.70 ± 5.49	No

**Table 4:** TBI-related factors associated with apathy

**Article characteristics**		**Apathy measure**	**TBI-related factors**		
**Article**	**Quality score**		**Factor**	**Mean ± SD**	**Significant effect**
(Andersson, 1999)		C-AES	Time since injury (months)	10.5 (SEM = 1.68)	No (TBI + A = TBI-A)
			Coma length (days)	8.2 (SEM = 1.64)	No (TBI + A = TBI-A)
(Andersson & Bergedalen, 2002)	6.5	C-AES	Time since injury (months)	12.2 ± 10.06	No
			Coma length (days)	9.2 ± 7.14	No
(Arnould et al., 2015)	6	Emotional blunting (C-AI)	Time since injury	38.85 ± 29.47	No
		Lack of initiative (C-AI)	Time since injury	38.85 ± 29.47	No
		Lack of interest (C-AI)	Time since injury	38.85 ± 29.47	No
(Arnould et al., 2018b)	9.75	I-IIS	Time since injury (months)	44.18 ± 38.85	No
		S-IIS	Time since injury (months)	44.18 ± 38.85	No
(Arnould et al., 2018a)	11.25	I-IIS	Time since injury	54.84 ± 60.25	No
			Initial Glasgow Coma Scale	7.1 (range: 3–14)	Yes (associated with IIS at 10-month follow-up)
(Glenn et al., 2002)		I-AES	TBI severity based on the Glasgow Coma Scale and loss of consciousness duration	18% moderate; 30% severe	No (moderate = severe)
(Knutson et al., 2014)	10.75	I-NPI	Level of consciousness at first examination	NR	No
			Duration of loss or alteration of consciousness	NR	No
(Lane-Brown & Tate, 2009a, b)		I-AES	Time since injury	80.58 ± 71.64	No
			Length of post-traumatic amnesia (days)	53.19 ± 43.62	No
		I-FrSBe	Time since injury	80.58 ± 71.64	No
			Length of post-traumatic amnesia (days)	53.19 ± 43.62	No
(Navarro-Main et al., 2021)		I-AES	Traumatic Axonal Injury No TAI Grade 1 Grade 2 Grade 3	47.5% 17.5% 15% 20%	No
			Injury Severity Score	33.7 (range: 16–50)	No
			Major Extracranial Injury (yes)	52.50%	No
			Glasgow Coma Scale – pre-hospital	8 (range: 3–15)	No
			Glasgow Coma Scale – admission	4.9 (range: 3–15)	No
			Glasgow Outcome Scale at discharge	3.2 (range: 2–5)	No
			Glasgow Coma Scale Extended at 6 months	5 (range: 3–8)	No
			Petechia (yes)	47.50%	No
			Contusion (yes)	45%	No
			Epidural hematoma (yes)	27.50%	No
			Subdural hematoma (yes)	47.50%	No

**Table 5:** Environmental factors of apathy

**Article characteristics**			**Environmental factors**			
**Article**	**Quality score**	**Apathy measure**	**Factor**	**Measure**	**Severity**	**Significant effect**
(Arnould et al., 2015)	6	Emotional blunting (C-AI)	Caregiver’s burden	Zarit Burden Interview	6.33 ± 4.28	Yes
		Lack of initiative (C-AI)	Caregiver’s burden	Zarit Burden Interview	6.33 ± 4.28	Yes
		Lack of interest (C-AI)	Caregiver’s burden	Zarit Burden Interview	6.33 ± 4.28	No
(Quang et al., 2021a)	11	I-DAS	Family functioning	The Family Assessment Device–General Functioning	26.61 ± 3.80	Yes (executive apathy and emotional apathy)
			Overprotectiveness of caregivers	The Overprotection Subscale of Questionnaire for Resources and Stress in Family	6.62 ± 2.54	Yes (executive apathy)

**Table 6:** Demographic factors associated with apathy

**Article characteristics**		**Apathy measure**	**Demographic factors**		
**Article**	**Quality score**		**Factor**	**Severity**	**Significant effect**
(Andersson & Bergedalen, 2002)	6.5	C-AES	Age	28.3 ± 9.17	No
			Education (years)	12.3 ± 2.38	No
			Sex (% Male)	75.50%	Yes (F < M)
(Arnould et al., 2018b)	9.75	I-IIS	Age	34.24 ± 12.52	No
			Education	13.39 ± 3.16	No
		S-IIS	Age	34.24 ± 12.52	Yes (*r* = 0.35)
			Education	13.39 ± 3.16	No
(Arnould et al., 2018a)	11.25	I-IIS	Age	34.78 ± 13.47	No
			Education	13.78 ± 3.16	No
(Lane-Brown & Tate, 2009a, b)		I-AES	Sex (% Male)	88.24	No
		I-FrSBe-A	Sex (% Male)	88.24	No
(Quang et al., 2021a)	11	I-DAS	Age	34.92 ± 11.86	No
			Education	8.87 ± 4.09	No
			Sex (% Male)	88.52	No

**Table 7:** Psychological factors of apathy

**Article characteristics**		**Apathy measure**	**Self-related and mood factors**			
**Article**	**Quality score**		**Factor**	**Measure**	**Mean ± SD**	**Significant effect**
(Arnould et al., 2018b)	9.75	I-IIS	Self-esteem	Rosenberg Self-Esteem Scale	30.53 ± 6.3	No
			Self-efficacy	General Self-efficacy Scale	26.61 ± 6.23	No
			Self-awareness	Patient Competency Rating Scale self-report	116.03 ± 20.1	No
				Patient Competency Rating Scale informant-report	111.47 ± 20.89	No
			Anxiety	Hamilton Depression Rating Scale	7.81 ± 4.72	No
			Depression		5.49 ± 4.03	No
		S-IIS	Self-esteem	Rosenberg Self-Esteem Scale	30.53 ± 6.3	Yes (*r* = − 0.62)
			Self-efficacy	General Self-efficacy Scale	26.61 ± 6.23	Yes (*r* = − 0.64)
			Self-awareness	Patient Competency Rating Scale self-report	116.03 ± 20.1	No
				Patient Competency Rating Scale informant-report	111.47 ± 20.89	No
			Anxiety	Hamilton Depression Rating Scale	7.81 ± 4.72	No
			Depression		5.49 ± 4.03	Yes (*r* = 0.62)
(Arnould et al., 2018a)	11.25	I-IIS	Self-esteem	Rosenberg Self-Esteem Scale	30.34 ± 6.59	Yes (*r* = − 0.39)
			Self-efficacy beliefs	General Self-efficacy Scale	26.03 ± 6.14	Yes (*r* = − 0.48)
			Anxiety	Hamilton Depression Rating Scale	7.94 ± 4.89	No
			Depression		6.07 ± 4.23	No
(Bivona et al., 2019)	10	I-NPI	Anxiety	State-Trait Anxiety Inventory – state anxiety	38.20 ± 11.91	No
				State-Trait Anxiety Inventory – trait anxiety	38.90 ± 13.15	No
			Depression	Hamilton Depression Rating Scale	9.63 ± 5.71	No
			Self-awareness	Patient Competency Rating Scale	3.17 ± 14.44	No
(Cristofori et al., 2019)	10.5	S-FrSBe	Loneliness	UCLA Loneliness (raw)	40.02 ± 10.60	Yes (*r* = 0.55)
		I-FrSBe		UCLA Loneliness (log-transformed)	1.59 ± 0.12	Yes (*r* = 0.29)
(Diaz et al., 2012)	9.5	S-AES	Personality changes	DSM-IV-TR	42.2 ± 17.3	Yes
(Knutson et al., 2014)	10.75	I-NPI	Pre-injury intelligence	Armed Forces Qualification Test	61.50 ± 25.54	No
			Depression	Beck Depression Inventory	9.16 ± 9.09	Yes (*r* = 0.24)
			Fatigue	Krupp’s Fatigue Severity	36.33 ± 11.92	No
			Depressive disorder	Structured Clinical Interview for DSM-IV-TR Axis I disorders, Non-Patient edition lifetime prevalence for major depressive disorder scores	1.40 ± 0.80	No
(Lane-Brown & Tate, 2009a, b)	9	I-AES	Depression	Depression, Anxiety and Stress Scale – Depression subscale	13.4 ± 12.5	No
			Fatigue	Barrow Neurological Institute Fatigue Scale	25.2 ± 19.5	No
		I-FrSBe-A	Depression	Depression, Anxiety and Stress Scale – Depression subscale	13.4 ± 12.5	No
			Fatigue	Barrow Neurological Institute Fatigue Scale	25.2 ± 19.5	No
(Quang et al., 2021a)	11	I-DAS	Self-efficacy	General Self-Efficacy scale	28.11 ± 5.24	Yes (initiation apathy)

### Meta-analysis Procedure

Three-level meta-analyses were carried out for the associations between apathy and cognitive dysfunction as well as between apathy and psychological variables, using random-effect models with a restricted maximum-likelihood estimator (Cheung, 2019). Three levels of variance were modelled: level 1 – sampling variance in effect size, level 2 – variance in effect sizes within studies and level 3 – variance in effect sizes between studies. To derive the overall correlation coefficient, Fisher’s *z* transformation was applied to the individual study findings and then back-transformed for reporting purposes. Such analyses were not possible for other factors due to the small number and methodological heterogeneity of studies. The analysis was performed using the metafor package in R Studio (version 4.3.1).

## Results

**Fig. 1:**
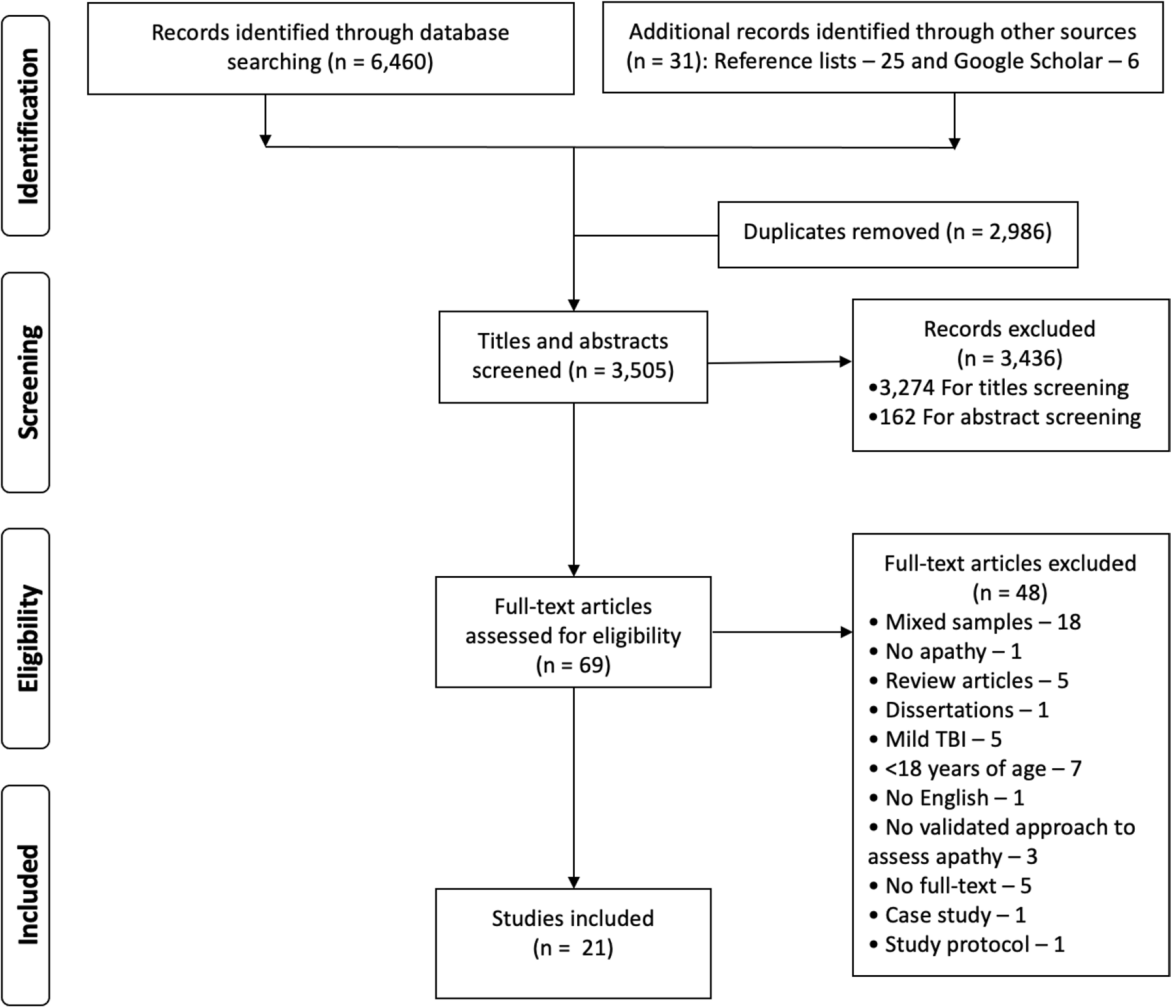
PRISMA flowchart with data on included and excluded studies

After abstract-title and full-text screening, 21 articles qualified for inclusion. The detailed process of literature search, screening process and exclusion of articles is presented in Fig. 1.

### Study Characteristics

Table 1 summarises details of the studies included in this review. In particular, they were carried out in the USA (*n* = 09), France (*n* = 04), Norway (*n* = 02), Spain (*n* = 02), Australia (*n* = 01), Brazil (*n* = 01), UK (*n* = 01) and Vietnam (*n* = 01). Of the 21 studies, 19 were cross-sectional and two were prospective. Sample sizes ranged from 30 to 176 participants and amassed to a total of sample of 1229 with an average age of 39.80 years. Participants with TBI were mainly males, but this proportion ranged from 64.4% to 100% across 20 studies (with the exception of one study with 11.9% males). The apathy measures varied from a self-report version (01/21), informant-reported versions (09/21), a combination of the two (08/21) or a clinician-reported version (03/21). The assessment tools used to measure apathy were the Apathy Evaluation Scale (AES; 05/21), Frontal Systems Behaviour Scale–Apathy Subscale (FrSBe-A; 05/21), Neuropsychiatric Inventory (NPI; 04/21), Initiative-Interest Scale (IIS; 03/21), Apathy Inventory (AI; 01/21), Dimensional Apathy Scale (DAS; 01/21), and the combination of the FrSBe-A with either The Lille Apathy Rating Scale (LARS and FrSBe-A; 01/21) or the Apathy Evaluation Scale (AES and FrSBe-A; 01/21). In terms of TBI severity, while 08/21 studies examined participants with moderate-to severe TBI, 11/21 studies included participants with only severe injuries and two studies included participants with penetrating injuries. Detail for time since injury was available in 16 studies, with the average around 4 years (58.85 months).

### Study Quality

The mean NIH quality assessment score of 21 papers was 9.5 (range from 6 to 12). Overall, the risk of bias of all papers was fairly low. Specifically, 8/19 cross-sectional studies were assessed at a low risk of bias (score from 10 to 13), 11/19 cross-sectional studies assessed at a moderate risk of bias (score 6–9.5) and no study assessed at a high risk of bias (score 0–4). Both longitudinal studies were evaluated at a moderate risk of bias. The lack of sample size justification, power description, or variance and effect estimates was the most common sources of bias that lowered the quality of those studies. Bias also occurred because TBI was not re-assessed/confirmed when the studies was conducted or because the participation rate of eligible people was not described. Another common source of bias in longitudinal studies was significant loss to follow-up after baseline.

### Prevalence and Severity of Apathy after TBI

Five studies investigated the prevalence of apathy and 12 examined the severity of apathy (Table 2). Overall, the prevalence of apathy varied between 31 and 72% with different cut-off scores used from separate apathy measures (Table 2). In two studies, 62.30 and 69% of patients with TBI were identified as having apathy based on the clinician version (cut-off ≥ 34) and the informant version (cut-off ≥ 37) of the AES, respectively (Andersson & Bergedalen, 2002; Lane-Brown & Tate, 2009a, b). On the FrSBe-A (*t*-score ≥ 65 for cut-off), the informant version showed 31 to 72% (Hogeveen et al., 2017; Lane-Brown & Tate, 2009a, b; Lengenfelder et al., 2015) and the self-report version determined 43% (Lengenfelder et al., 2015) as the prevalence of apathy after TBI. Based on the clinician version of the NPI, 49% of the patients with TBI were found to have apathy (Monsalve et al., 2012).

Of 12 studies that reported apathy severity after TBI, 5 included neurologically healthy participants for comparison. All five studies demonstrated higher levels of apathy in the TBI compared to a healthy comparison group (Arnould et al., 2018b; De Simoni et al., 2018; Hart et al., 2019; Hogeveen et al., 2017; Quang et al., 2021a), except that one study did not find significant impairment in executive apathy and emotional apathy following TBI using the self-reported DAS (Quang et al., 2021a).

### Factors Associated with Apathy after TBI

#### Neurobiological Factors

Patients with TBI typically have multi-focal lesions evident from brain scans, leading to enormously heterogeneous phenotypes across patients. However, the prefrontal lobes and their connectivities represent the most vulnerable and commonly affected regions, often resulting in deficits in executive functions essential for cognitive and behavioural regulation, and drive (Bigler, 2001; Haber, 2016; McAllister, 2011; Rabinowitz & Levin, 2014). Of importance here, disruptions to the prefrontal-subcortical circuits have also consistently been found to be neural correlates of apathy across different neurological disorders (Kos et al., 2016; Le Heron et al., 2018; Levy & Dubois, 2006; Quang et al., 2021b), and therefore may be relevant to apathy following TBI. Cognitive dysfunction is also a helpful index of neuronal pathway disruptions associated with apathy. Finally, injury-related factors such as injury severity and post-traumatic amnesia have also been shown to predict behavioural and functional outcomes in patients with TBI (Ponsford, 2013).

#### Neural Disruptions

**Fig. 2:**
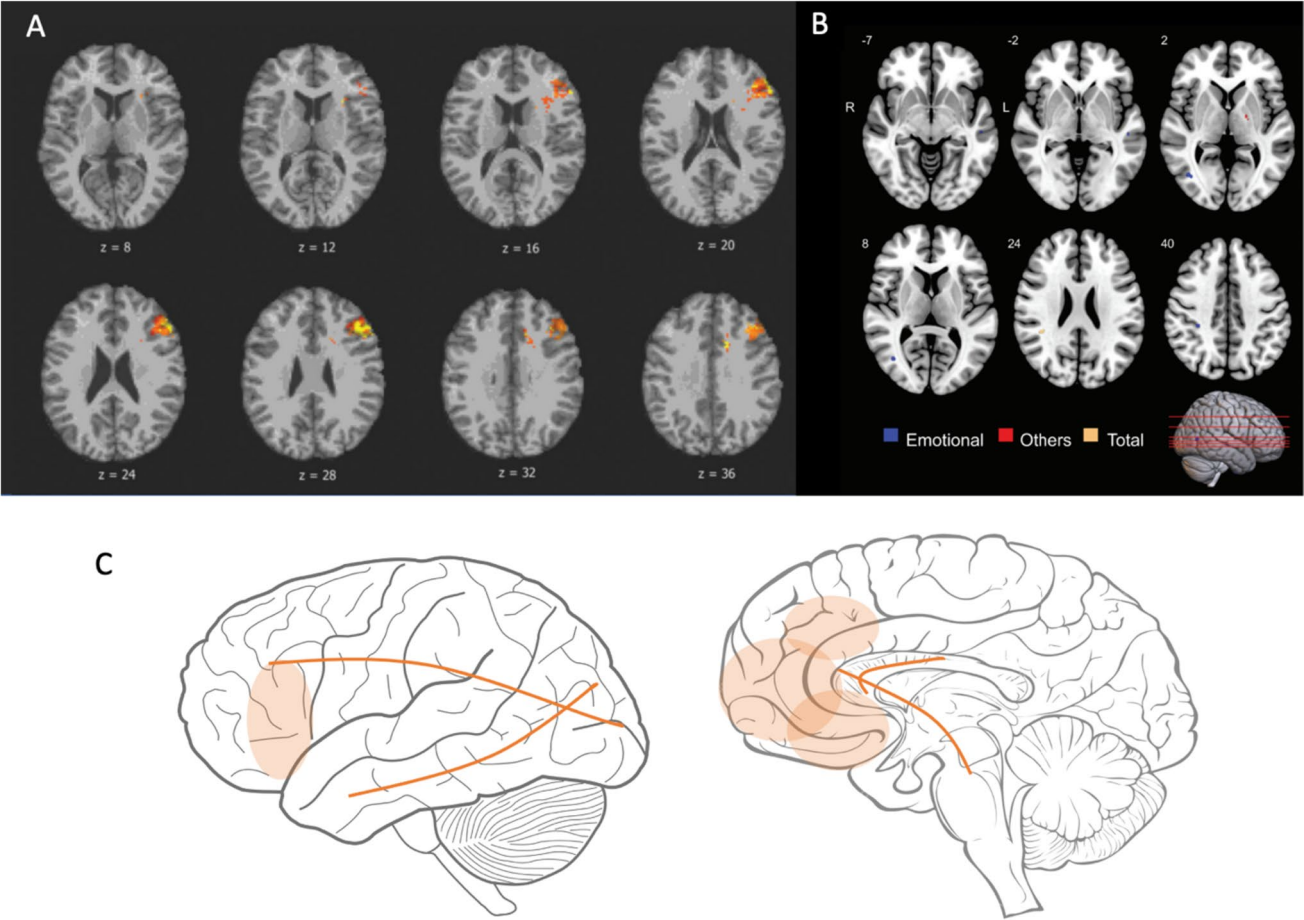
Neural correlates of apathy after moderate-to-severe TBI. **A** The left frontal lobe, anterior cingulate cortex, insula, supplementary motor cortex related to apathy in the Knutson et al. (2014) study. **B** Reduced FA in the inferior longitudinal fasciculus, the superior longitudinal fasciculus and the posterior limb of internal capsule correlated with increased apathy in the Navarro-Main et al. (2021) study. **C** A summary of reviewed evidence for the neural correlated associated with apathy. *Note.* Images adapted with permission from Oxford University Press and Taylor & Francis

Four studies (De Simoni et al., 2018; Hogeveen et al., 2017; Knutson et al., 2014; Navarro-Main et al., 2021) included in this review investigated neural correlates of apathy after TBI, with two focusing on penetrating TBI and two on closed TBI. Comparing well-characterised groups of TBI (patients with damage to the ventromedial prefrontal cortex, patients with damage to the dorsomedial prefrontal cortex, patients with damage to other brain regions) and controls, Hogeveen et al. (2017) found greater severity and a higher prevalence of apathy on the informant-rated FrSBe-A in patients with damage to the ventromedial prefrontal cortex compared to the others. Importantly, the effect of ventromedial frontal damage in apathy was mediated through impaired stimulus valuation assessed with the Becker–DeGroot–Marschak auction task. Also, researching patients with penetrating TBI, Knutson et al. (2014) demonstrated that apathy symptoms measured with the informant-rated NPI were correlated with grey matter damage to the left frontal lobe, anterior cingulate cortex, insula, supplementary motor cortex (Fig. 2A) and white matter damage to the superior and anterior corona radiata, and the genus and body of the corpus callosum.

A study of patients with severe closed head injury found that reduced fractional anisotropy (FA: a neuroimaging measure of white matter function) of the superior longitudinal fasciculus was associated with total I-AES and Emotional I-AES (Navarro-Main et al., 2021). Furthermore, reduced FA of the inferior longitudinal fasciculus was associated with emotional apathy on the informant-rated AES only, and reduced FA of the posterior limb of internal capsule was associated with apathy on the informant-rated AES only (Fig. 2B) (Navarro-Main et al., 2021). White matter tracts within the subcortical structures were also affected (Navarro-Main et al., 2021). Research by De Simoni reported that increased connectivity of the left anterior caudate was associated with increased apathy on the self-reported FrSBe, although the relationship became non-significant after correcting for multiple comparisons (De Simoni et al., 2018). In summary, current evidence for neurobiological underpinnings of apathy remains limited. The evidence suggests that apathy after TBI is associated with grey matter changes in not only the prefrontal cortex, but also the widespread structures of the frontal lobes including the anterior cingulate cortex, the insula and supplementary motor areas (Fig. 2C). White matter alterations related to apathy after TBI are seen in the bundles that connect the frontal lobes with subcortical and posterior structures.

#### Cognitive Dysfunction

Cognitive dysfunction associated with apathy was reported in nine studies (Table 3). Two of them evaluated *general cognitive dysfunction* (with one using overall scores of MoCA and one reporting scores for verbal and non-verbal skills on the WAIS) and found no significant relationships with apathy (Andersson & Bergedalen, 2002; Quang et al., 2021a). On the other hand, there was some suggestion of a specific relationship between apathy and *learning*, although results were mixed. Learning difficulty was moderately associated with apathy assessed with the clinician version of the AES and the informant reported IIS (Andersson & Bergedalen, 2002; Arnould et al., 2018b). Importantly, learning difficulties identified by the California Verbal Learning Test at baseline significantly predicted apathy on the informant-version IIS 10 months later (Arnould et al., 2018a). However, learning was not related to apathy as measured with the self-reported IIS, the informant-reported AES and the informant-reported FrSBe-A (Arnould et al., 2018b; Lane-Brown & Tate, 2009a, b). There was fairly consistent evidence that *attention/working memory* did not predict apathy (Andersson & Bergedalen, 2002; Arnould et al., 2018a, b; Knutson et al., 2014; Lane-Brown & Tate, 2009a, b; Lengenfelder et al., 2015), with only one study finding a negative, moderate correlation between the Digit Span test – scaled scores and the self-reported FrSBe-A scores (Lengenfelder et al., 2015). Similarly, *reduced information* and *motor processing speed* were consistently reported as not relating to apathy (Andersson & Bergedalen, 2002; Arnould et al., 2018a, b).

In terms of executive dysfunctions, *planning and multitasking* difficulties were reported with mixed findings by two papers, which likely consisted of overlapping samples. Particularly, Arnould et al. (2018b) reported an insignificant relationship between the Meeting Preparation Task inefficiency scores and both the self-report and informant rated IIS scores (*n* = 38), whereas Arnould et al. (2018a, b, c) demonstrated a borderline significant prediction of the Meeting Preparation Task inefficiency scores for the informant-rated IIS (*n* = 34). Furthermore, inefficiency on this task mediated the relationship between verbal episodic learning and apathy on the IIS (Arnould et al., 2018c). In a follow-up study, the Meeting Preparation Task inefficiency scores at baseline, however, did not predict the IIS scores 10 months later (Arnould et al., 2018a). Mixed findings were found for *idea generation* and *flexibility*. Lower performance on the Wisconsin Card Sorting Test was linked to lower apathy scores, as reported by two studies (Andersson & Bergedalen, 2002; Bivona et al., 2019). By contrast, investigations by Lengenfelder et al. (2015) and Lane-Brown and Tate (2009a, b) did not support the association between scores on the Verbal Fluency, Wisconsin Card-Sorting Test, Thurstone Word Fluency Test and apathy scales such as FrSBe-A or AES.

**Fig. 3:**
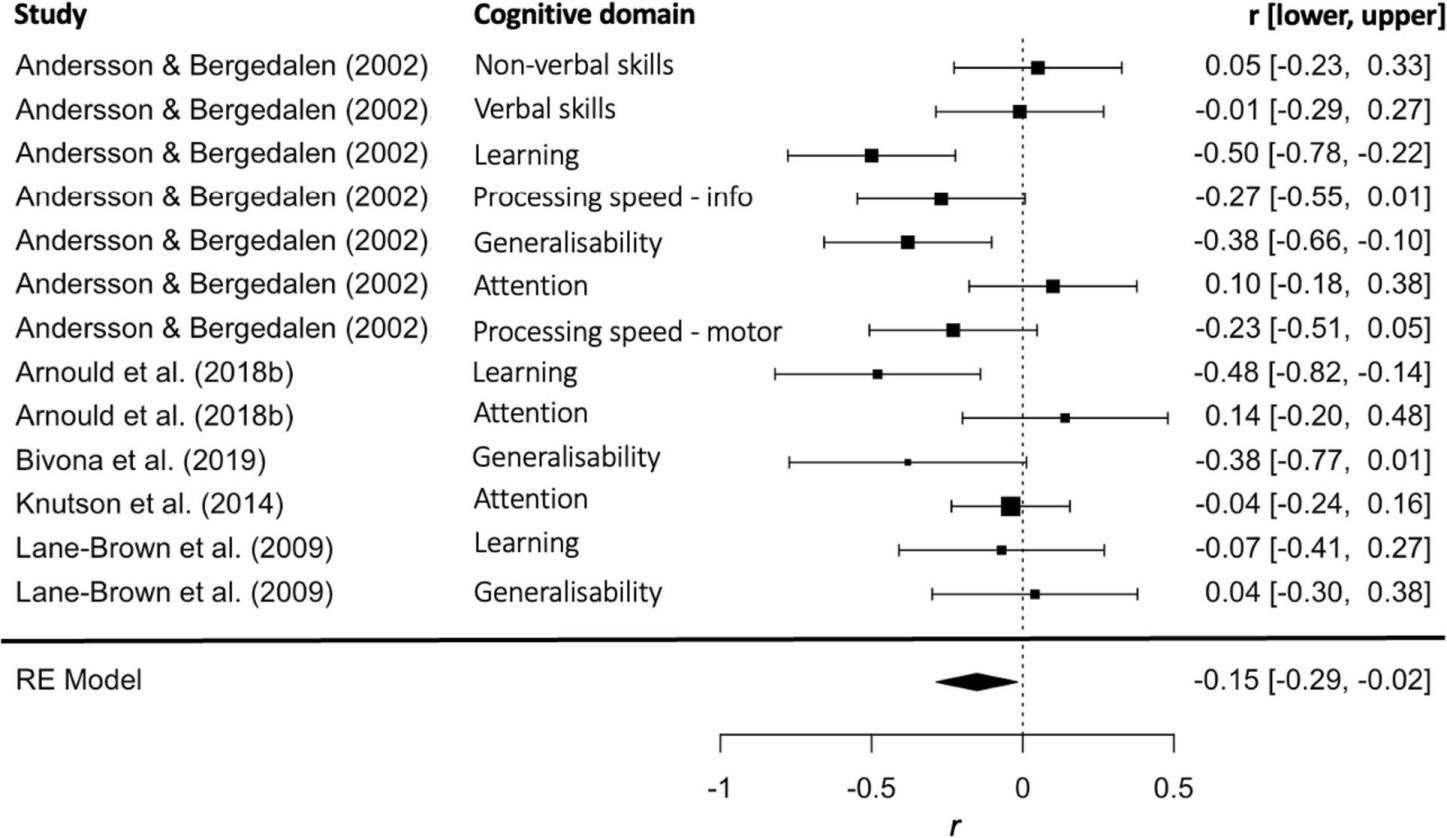
Correlational meta-analysis for the association between apathy and cognitive functions. Effect sizes are depicted with 95% CI. The size of the square indicates the relative sample size of each study (and weight assigned in the meta-analysis). The overall effect size (and 95% CI) is represented with the diamond

After excluding data that were duplicated and/or derived from heterogenous methods, 13 datasets were entered in the correlational meta-analysis. Figure 3 shows that the overall correlation between cognitive functions and apathy was small but significant across studies (Fisher’s* z* = − 0.163 equivalent to *r* = − 0.162, *p* < 0.05). Assessment of heterogeneity was carried out, with *Q*(12) = 29.94, *p* < 0.003. The estimated variance components were σ^*2*^_*Level 2*_ = 0.00 and σ^*2*^_*Level 3*_ = 0.33*.* Particularly, 39.39% of the overall variance was attributed to level 1, 0% to level 2 (within-study variance) and 60.12% to level 3 (between-study variance). Subgroup assessment was not possible due to insufficient data.

#### TBI-Related Factors

As shown in Table 4, TBI-related factors investigated were (1) TBI severity including coma length, Glasgow Coma Scale scores, length of post-traumatic amnesia and level of consciousness; (2) TBI characteristics such as traumatic axonal injury, presence of major extracranial injury, contusion, petechia, epidural hematoma and subdural hematoma; and (3) time since injury. These factors were consistently reported as not correlated with apathy (Andersson & Bergedalen, 2002; Andersson et al., 1999; Arnould et al., 2015, 2018b; Glenn et al., 2002; Knutson et al., 2014; Lane-Brown & Tate, 2009a, b; Navarro-Main et al., 2021). Only one longitudinal study demonstrated a relationship between lower initial Glasgow Coma Scale and greater levels of apathy around 5 years since the injury (Arnould et al., 2018a).

#### Socio-environmental Factors

Although the primary underpinning of apathy is neurobiological, there is evidence that the patient’s social and environmental factors play a crucial role in functional outcomes (Ponsford, 2013; Whiteneck et al., 2004; Wong et al., 2017). These external factors can range from physical environment in which the person with TBI lives through to their socio-cultural context, involving knowledge and attitudes of the people interacting with them (Whiteneck et al., 2004). Theoretically, these factors are important mediators of apathy as they indicate the degree and source of potential rewards available for activities as well as guidance and expectations which can either facilitate or undermine dependence and goal-directed actions in people with TBI (Marin, 1991).

Surprisingly, only two studies investigated social and environment factors of apathy (Arnould et al., 2015; Quang et al., 2021a) (Table 5). Arnould et al. (2015) showed that greater perceived burden of caregivers (as assessed with the Zarit Burden Interview) was significantly correlated with higher levels of two apathy dimensions: lack of initiative and emotional blunting on the clinician-version AI. Although the authors interpreted the results as indicating the impact of apathy on the carer’s burden and distress, it was also likely that such heightened carer burden could trigger or exacerbate the apathy symptoms in the patients. No relationship, however, between lack of interest as a dimension of apathy and caregiver burden was found.

Quantitative comparison of severity and prevalence of apathy across cultures is beyond the scope of this study. However, when considering the distribution of research across cultural groups, there is a skew towards Western and individualistic countries. Only one study has been conducted in an Eastern society – Vietnam (Quang et al., 2021a). This is also the only investigation that considered cultural factors associated with apathy. In consideration of the specific collective cultural features of Vietnam, Quang et al. (2021a, b) identified two family factors that might be associated with apathy in Vietnamese individuals with TBI: family functioning and overprotective behaviour of caregivers. Indeed, these two factors were found to relate to executive and emotional symptoms of apathy. Particularly, unhealthy family functioning and more overprotectiveness of carers was associated with higher levels of executive and emotional apathy (Quang et al., 2021a).

#### Individual Factors

Individual factors are identified as the physical and psychological influences that are specific to the patient. They may include their genetic features, demographic characteristics, and premorbid physical and psychological status. Emerging evidence has suggested a contributing role of these combined personal factors in functional and behavioural outcomes after TBI (Ponsford, 2013).

In this review, 11 studies examined patients’ individual factors and their influence on apathy. *Demographic variables* were not found to correlate with apathy in general, as reported by most of the studies (Andersson & Bergedalen, 2002; Arnould et al., 2018a, b; Lane-Brown & Tate, 2009a, b; Quang et al., 2021a) (Table 6). Only Arnould et al. (2018b) found a positive correlation between age and apathy on the self-version of the Initiative-Interest Scale, and Andersson and Bergedalen (2002) showed significantly lower levels of apathy in females compared to males with TBI.

Only one study (Knutson et al., 2014) evaluated *premorbid psychological features* in patients. The study found that *pre-injury intelligence* was not associated with apathy symptoms (Table 7). Given that *post-injury psychological* variables might be influenced by the pre-injury levels, we included the reported findings here (also see Table 7). Two studies investigated the relationship between apathy and fatigue (Knutson et al., 2014; Lane-Brown & Tate, 2009a, b). Neither of them found that fatigue was associated with apathy. Five studies investigated the links between apathy and mood (Arnould et al., 2018a, b; Bivona et al., 2019; Knutson et al., 2014; Lane-Brown & Tate, 2009a, b). None of them showed significant correlations between apathy and anxiety (Arnould et al., 2018a, b; Bivona et al., 2019; Lane-Brown & Tate, 2009a, b). Evidence for the relationship between depression and apathy was inconsistent. Apathy was correlated with depression on the self-reported HADS (Arnould et al., 2018b) and informant-reported BDI (Knutson et al., 2014). By contrast, the association between apathy and depression was not observed when depression was measured by DASS – Depression subscale (Lane-Brown & Tate, 2009a, b), informant-version of the HADS (Arnould et al., 2018a; Bivona et al., 2019) or the Structured Clinical Interview for DSM-IV-TR (Knutson et al., 2014). However, the patients’ perceived loneliness was a factor relating to apathy, with greater loneliness on the UCLA Loneliness scale associated with heightened apathy measured by both the informant-reported and patient-reported versions of FrSBe-A (Cristofori et al., 2019).

Self-related factors of apathy including self-efficacy, self-esteem, personality changes and self-awareness were investigated in four studies (Arnould et al., 2018a, b; Bivona et al., 2019; Quang et al., 2021a). Overall, the relationship between greater self-efficacy and lower apathy, especially the initiation dimension, was demonstrated by most of the studies (Arnould et al., 2018a, b; Bivona et al., 2019; Quang et al., 2021a). While apathy assessed with the informant-rated IIS was not linked to self-efficacy at baseline, the association was observed at 1-year follow-up (Arnould et al., 2018a). Similarly, higher apathy was only related to lower self-esteem when examined with the self-reported measure of the IIS at baseline (Arnould et al., 2018b), while the relationship emerged for the informant report IIS at 1-year follow-up (Arnould et al., 2018a). The only study that assessed apathy in relation to personality change found that greater apathy significantly correlated with more personality changes as measured with the DSM-IV-TR (Diaz et al., 2012). Self-awareness, by contrast, was not related to apathy symptoms, as reported by two studies (Arnould et al., 2018b; Bivona et al., 2019).

**Fig. 4:**
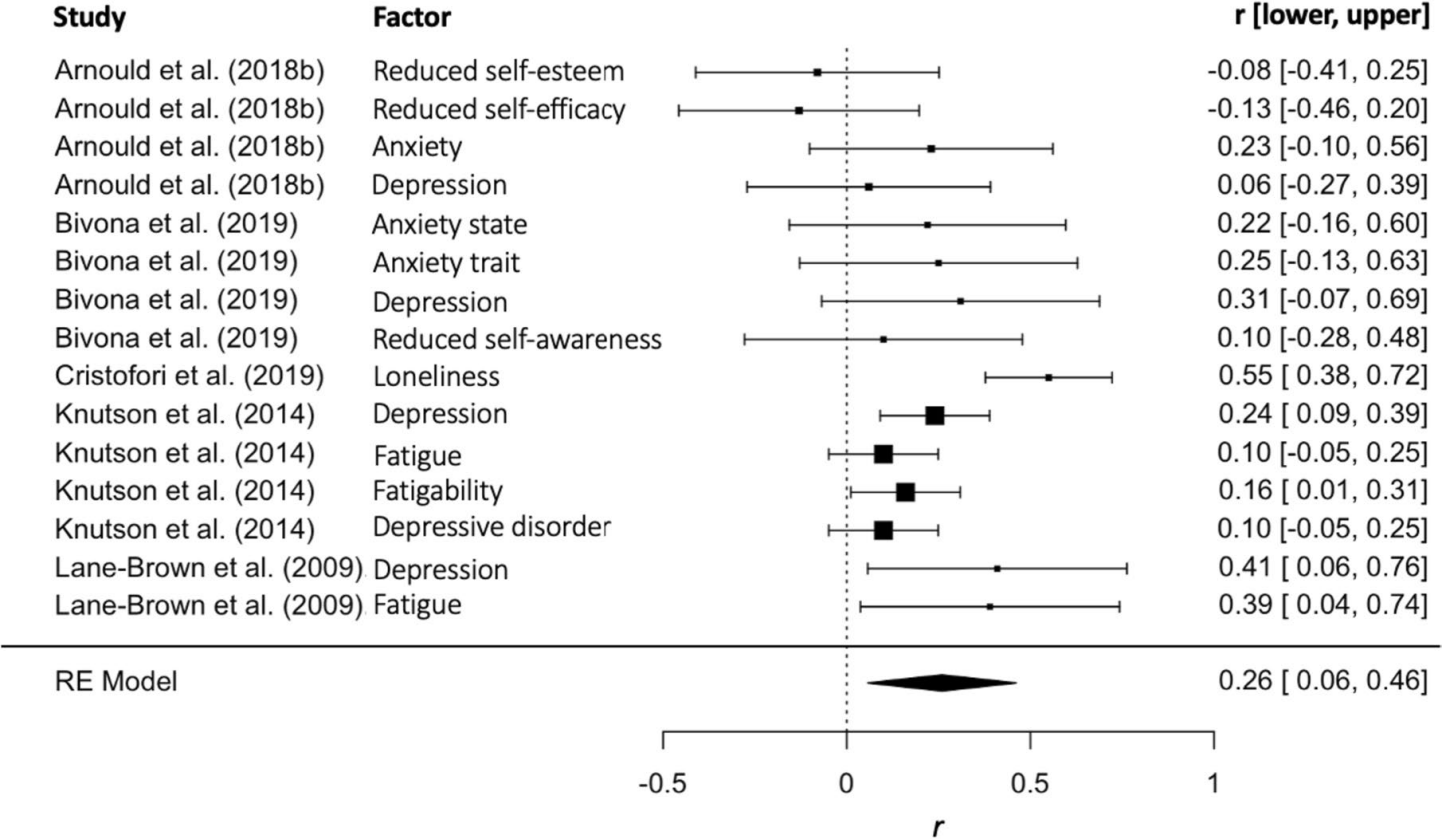
Correlational meta-analysis for the association between apathy and psychological factors. Effect sizes are depicted with 95% CI. The size of the square indicates the relative sample size of each study (and weight assigned in the meta-analysis). The overall effect size (and 95% CI) is represented with the diamond

After excluding data that were duplicated and/or derived from heterogenous methods, the correlational meta-analysis included 15 datasets. Figure 4 shows that the overall correlation between apathy and psychological factors was small but significant across studies (Fisher’s* z* = 0.30 equivalent to *r* = 0.291, *p* < 0.01). Heterogeneity was evaluated, with *Q*(14) = 31.29, *p* = 0.005. The estimated variance components were σ^*2*^_*Level 2*_ = 0.037 and σ^*2*^_*Level 3*_ = 0.00*.* Specifically, 27.37% of the overall variance was attributed to level 1, 72.62% to level 2 (within-study variance) and 0% to level 3 (between-study variance). The sample sizes were too small for subgroup analyses.

## Discussion

Apathy is commonly reported after moderate-to-severe TBI, with prevalence rates ranging from 31 to 72%, as reviewed by this synthesis. The main aim of this systematic review and meta-analysis was to examine evidence on neurobiological, socio-environmental and individual factors of apathy after moderate-to-severe TBI, based on the biopsychosocial model. We found that the majority of work has focused on cognitive dysfunctions, TBI-related factors, demographic variables and psychological correlates of apathy, while evidence for neural substrates and socio-cultural and premorbid aspects is scant. Overall, the current knowledge suggests that demographic and TBI-related factors did not contribute to apathy, whereas complex neurocognitive alterations, socio-environmental and cultural context as well as patients’ self-related factors may be important components of this motivational disorder arising after TBI.

Results for neurobiological factors are drawn heavily from TBI variables and cognitive dysfunctions. A relative consensus of findings across studies is that TBI factors such as time since injury and TBI severity measured via the Glasgow Coma Scale and post-traumatic amnesia (Andersson & Bergedalen, 2002; Andersson et al., 1999; Arnould et al., 2015, 2018b; Glenn et al., 2002; Knutson et al., 2014; Lane-Brown & Tate, 2009a, b; Navarro-Main et al., 2021) were not associated with apathy. These findings suggest that apathy persists irrespective of the severity of TBI or time since injury, which provides evidence against the assumptions that apathy can only be seen in patients with acute TBI. However, caution should also be noted when interpreting these results as the Glasgow Coma Scale or post-traumatic amnesia alone may not always be reliable measures of TBI severity. Additional assessment such as duration of loss of consciousness, neuroimaging studies and neurological investigations would be helpful for future studies to accurately identify the extent of injury.

The correlation meta-analysis indicated an overall significant relationship between apathy and cognitive dysfunction, although a subgroup meta-analysis for each cognitive domain was not possible. The qualitative synthesis showed that apathy was not associated with attention/working memory, information and motor processing speed (Andersson & Bergedalen, 2002; Arnould et al., 2018a, b; Knutson et al., 2014; Lane-Brown & Tate, 2009a, b; Lengenfelder et al., 2015). Although deficits of attention and processing speed are well documented after TBI (Mathias & Wheaton, 2007), our review suggests they are independent from apathy – the impairment of goal-directed disorder. Some evidence was reported for the association between increased apathy and learning and executive functioning difficulties, such as planning, multitasking, idea generation and flexibility (Andersson & Bergedalen, 2002; Arnould et al., 2018b; Bivona et al., 2019; Lane-Brown & Tate, 2009a, b). Notably, these findings are in line with the sporadic neuroimaging evidence synthesised in this review. These neuroimaging results suggests that apathy after TBI is associated with neural changes in the widespread structures of the frontal lobes including the prefrontal cortex, anterior cingulate cortex, insula and supplementary motor areas (De Simoni et al., 2018; Hogeveen et al., 2017; Knutson et al., 2014; Navarro-Main et al., 2021). Together, these results suggest the possible organic nature of apathy, such that apathy specifically arises from the neural disruptions of executive function and reward processing pathways, which has been demonstrated across different neurological conditions such as stroke, Parkinson’s disease, Alzheimer’s disease and frontotemporal dementia (Kumfor et al., 2018; Le Heron et al., 2019; Quang et al., 2021b). However, more neuroimaging research in TBI is warranted.

Very limited evidence was reported for the socio-environmental factors of apathy after moderate-to-severe TBI. The existing work found that greater carer burden, more overprotective behaviour in carers and unhealthy changes to family functioning were related to higher levels of apathy in the patients with TBI (Quang et al., 2021a). Regarding individual factors, a general agreement is observed for the lack of association between apathy after TBI and demographic features (such as age, education and sex). Our meta-analysis showed an overall effect for the relationship between apathy and psychological variables, suggesting that higher levels of apathy are associated with greater psychological issues after TBI. Through systematic synthesis, dissectible profiles emerged for different psychological factors. Specifically, while apathy may not be related to pre-injury intelligence, fatigue, anxiety and self-awareness (Andersson & Bergedalen, 2002; Arnould et al., 2018a, b; Bivona et al., 2019; Knutson et al., 2014; Lane-Brown & Tate, 2009a, b; Quang et al., 2021a), it is likely to link with loneliness and self-related characteristics such as lower self-efficacy, lower self-esteem and more personality changes (Arnould et al., 2018a, b; Bivona et al., 2019; Diaz et al., 2012; Quang et al., 2021a). As such, these initial findings underscore that apathy is beyond the impairment of neural networks and has complex environmental-individual interactions.

Our results particularly shed light on the extent to which apathy and self-efficacy are tautological. While the definitions of apathy and self-efficacy are distinct, it is possible that patients’ perceptions when answering items related to these constructs may overlap, which can lead to a tautological relationship. This only occurs when both self-efficacy and apathy measures are rated by the patients themselves. In such cases, our results indeed indicated a strong association (*r* = − 0.64). However, it is important to note that most of the studies reviewed in this paper assess self-efficacy via self-report and assess apathy by informant ratings, or information ratings and self-report. This helps to reduce the potential tautological issues between the two constructs, as the informant may have a more objective perspective on the patient’s behaviour and level of motivation. For meta-analyses, we prioritised informant-report measures of apathy over patient self-report in studies that used the two versions, due to the potential lack of insight in patients and the risk of tautological issues between the two constructs. Therefore, while there may be some overlap in patients’ perceptions of these constructs, the use of informant-report measures can help to differentiate between apathy and self-efficacy more effectively.

Collectively, our review highlights the importance of the biopsychosocial approach in clinical practice. The depth of apathy assessment after moderate-to-severe TBI can be increased by not only understanding symptom subtypes and different aspects of medical issues such as the nature of injury and what neuronal structures and pathways are affected, but also the patients’ pre-injury characteristics as well as what has happened to the patients on both environmental and individual levels after the injury. Another vital aspect that healthcare providers can consider is the appropriateness of assessment tools. Preferences should be given to measures that are not only translated but also validated and congruent to the patients’ cultures as those tools will provide more accurate and valuable information (Arafat et al., 2016; Quang et al., 2022b). Interventions and treatment for apathy should also be built on both pharmacological and psychosocial components, when necessary. Of importance, psychoeducation for both patients and carers may be crucial for providing consistently appropriate stimuli and support. When patients and carers are aware that apathy is not a behavioural problem that is entirely attributed to neural disruptions but can be malleable, they can develop plans and strategies to modify their own environment and overcome social barriers.

From a research perspective, there are three main recommendations for future directions. First, adoption of the biopsychosocial approach to examine the interaction between factors is needed in future work to provide evidence for the development of systematic guidelines that tailor assessment and interventions of behavioural impairments, including apathy, for individual clients with moderate-to-severe TBI. Second, more advanced research is warranted regarding the neurobiological, socio-environmental and individual contributions to apathy. Particularly, for better understanding of cognitive mechanisms underpinning apathy, the use of single neuropsychological tests may not be sufficient, as reflected in the mixed results seen in the review. Rigorous behavioural experiments grounded on well-developed theories may be important to assess the complex cognitive processes underlying apathy following TBI (Morris et al., 2022; Quang et al., 2022a, 2023). In terms of socio-environmental factors, assessing features that are specific to the individual’s cultural context has the potential to facilitate translational applications of research. Cultural values, such as collectivism and individualism, are important as they can influence family/carer behaviour and shape social norms for what to expect in people with TBI (Roy et al., 2015). For example, in some cultures, people with brain-related conditions are perceived as having madness and/or should withdraw from life and, therefore, opportunities for those people to engage in social activities become less available (Nguyen & Li, 2020; Simpson et al., 2000). Moreover, research in individual factors needs to account for pre-morbid levels, rather than focusing predominantly on psychological factors that are measured at the same time as apathy post-injury, so that individual variability can be better understood. Finally, most of the studies identified in this review employed cross-sectional rather than longitudinal designs, making it is unclear whether apathy is caused by a psychosocial factor or if a third factor is responsible for both apathy and the psychosocial factor. Therefore, longitudinal investigations are warranted to determine the causality of relationships. Prospective longitudinal studies may also provide a more in-depth understanding of the adjustment process and clearer guidance concerning the changing support needs of individuals and their families.

In conclusion, apathy is a disruptive sequala of moderate-to-severe TBI. Evidence for factors associated with apathy following moderate-to-severe TBI has now been systematically reviewed for the first time. The evidence points to the complex interplay of neural disturbances in the prefrontal cortex, socio-environmental and individual factors underlying apathy, while suggesting that TBI-related factors and patient’s basic demographics are less influential in apathy presentations. Moving forward, studies should investigate the complex interaction of impairments in cognitive processing as well as cultural and pre-injury characteristics to better understand the aetiology of apathy. This would greatly enhance the future development of effective intervention and management of this detrimental behavioural deficit following moderate-to-severe TBI.

## Supplementary Information

Below is the link to the electronic supplementary material.

Supplementary file1 (DOCX 17 KB)

## Data Availability

Not available.
