# Supplementary material for: A Biopsychosocial Framework for Apathy Following Moderate to Severe Traumatic Brain Injury: A Systematic Review and Meta-analysis
Source: Neuropsychol Rev. 2023 Dec 19;34(4):1213–34. doi: 10.1007/s11065-023-09620-4 (PMC11607053; doi:10.1007/s11065-023-09620-4)
Supplement: Supplementary file 1 — Supplementary file1 (DOCX 17 KB) [file 11065_2023_9620_MOESM1_ESM.docx]

**MODIFIED NIH QUALITY ASSESSMENT SCALE FOR CROSS-SECTIONAL AND COHORT STUDIES**

| **Criteria** | **Yes/No** | **Specify** |
| --- | --- | --- |
| 1. Was the research question or objective in this paper clearly stated?  Yes: Research questions were clearly stated.  No: Research questions were hard to understand. No research questions stated. |  |  |
| 2. Was the study population clearly specified and defined?  Yes: Demographics, location, and time period were described.  No: No description. |  |  |
| 3. Was the participation rate of eligible persons at least 50%?  Yes: The participation rate of eligible persons was at least 50%  No: The participation rate of eligible persons was higher than 50%. No description. |  |  |
| 4. Were all the subjects selected or recruited from the same or similar populations (including the same time period)? Were inclusion and exclusion criteria for being in the study prespecified and applied uniformly to all participants?  Yes: Same population, same inclusion and exclusion criteria across participants.  No: Different population, inclusion and exclusion criteria. No description. |  |  |
| 5. Was a sample size justification, power description, or variance and effect estimates provided?  Yes: A sample size justification, power description, or variance and effect estimates was provided.  No: No description |  |  |
| 6. Ensure apathy did not occur before TBI.  Yes: Exclusion criteria for no neurological, psychiatric disorders or prior TBI was included. Pre-injury apathy level was controlled.  No: No description. |  |  |
| 7. Was the timeframe sufficient and valid so that one could reasonably expect to see an association between exposure and outcome if it existed?  Yes: Time since injury was at least 6 months  No: Time since injury was less than 6 months. No description. |  |  |
| 8. For exposures that can vary in amount or level, did the study examine different levels of the exposure as related to the outcome (e.g., categories of exposure, or exposure measured as continuous variable)?  Yes: Severity of TBI was clearly described.  No: No description. |  |  |
| 9. Were the exposure measures (independent variables) clearly defined, valid, reliable, and implemented consistently across all study participants?  Yes: TBI was identified based on medical record (e.g., GCS, PTA, and brain scan).  No: TBI was identified based on self-report. |  |  |
| 10. Was the exposure(s) assessed more than once over time? (for cohort studies)  Yes: TBI was assessed more than once over time  No: TBI was not assessed more than once over time. No description. |  |  |
| 11. Were the outcome measures (dependent variables) clearly defined, valid, reliable, and implemented consistently across all study participants?  Yes: Validated apathy assessment tools were used  No: non-validated apathy assessment tools. No description. |  |  |
| 12. Were the outcome assessors blinded to the exposure status of participants?  Yes: A self-report, informant version or clinical version (where the assessor did know the research question) of apathy questionnaires was included. Behavioural tasks. Physiological record.  No: Structured interview or report where the interviewer knows who in which group. No description. |  |  |
| 13. Was loss to follow-up after baseline 20% or less? (for cohort studies)  Yes: More than 20%  No: 20% or less. No description. |  |  |
| 14. Were key potential confounding variables measured and adjusted statistically for their impact on the relationship between exposure(s) and outcome(s)?  Yes: Use clearly described and appropriate statistics controlling for confounding variables, present the measurement of association and include confidence intervals and/or the probability level (p value)  No: Use inappropriate statistical tests. Did not control for confounding variables. Did not present confidence intervals and/or the probability level (p value) of results. No description. |  |  |
| 15. Was a control group included and matched for age, education and gender with TBI group?  Yes: A control group was included and matched for age, education and gender with TBI group. An apathy assessment tool with standardised norm stratified for age, education and gender was used for analysis.  No: No control group or apathy assessment tool with standardised norms. |  |  |
